# [*N*′-(5-Bromo-2-oxidobenzyl­idene-κ*O*)-3-hydr­oxy-2-naphthohydrazidato-κ^2^ *N*′,*O*]dicyclo­hexyl­tin(IV)

**DOI:** 10.1107/S1600536810001145

**Published:** 2010-01-16

**Authors:** See Mun Lee, Hapipah Mohd Ali, Kong Mun Lo

**Affiliations:** a Department of Chemistry, University of Malaya, 50603 Kuala Lumpur, Malaysia

## Abstract

The environment at the Sn^IV^ atom in the title compound, [Sn(C_6_H_11_)_2_(C_18_H_11_BrN_2_O_3_)], is distorted trigonal-bipyramidal, with the two cyclo­hexyl groups and the imino N atom forming the equatorial plane. The axial O—Sn—O angle is 155.97 (9)°. The presence of an intra­molecular O—H⋯N hydrogen bond in the Schiff base ligand helps to stabilize the overall structure.

## Related literature

For related structures, see Lee *et al.* (2009*a* ▶,*b* ▶). For related dianions of similar hydrazone *O,N,O′*-chelates to tin in organotin compounds, see: Labib *et al.* (1996 ▶); Samanta *et al.* (2007 ▶).
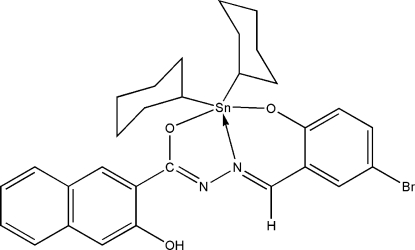


## Experimental

### 

#### Crystal data

[Sn(C_6_H_11_)_2_(C_18_H_11_BrN_2_O_3_)]
*M* *_r_* = 668.18
Monoclinic, 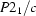

*a* = 9.2442 (2) Å
*b* = 9.9949 (2) Å
*c* = 29.6493 (5) Å
β = 94.874 (1)°
*V* = 2729.54 (9) Å^3^
*Z* = 4
Mo *K*α radiation
μ = 2.43 mm^−1^
*T* = 140 K
0.33 × 0.30 × 0.14 mm

#### Data collection

Bruker APEXII CCD area-detector diffractometer
Absorption correction: multi-scan (*SADABS*; Sheldrick, 1996 ▶) *T*_min_ = 0.501, *T*_max_ = 0.727
20482 measured reflections
4787 independent reflections
4103 reflections with *I* > 2σ(*I*)
*R*_int_ = 0.027

#### Refinement

*R*[*F* ^2^ > 2σ(*F* ^2^)] = 0.035
*wR*(*F* ^2^) = 0.079
*S* = 1.09
4787 reflections
335 parameters
1 restraint
H-atom parameters constrained
Δρ_max_ = 1.60 e Å^−3^
Δρ_min_ = −0.49 e Å^−3^

### 

Data collection: *APEX2* (Bruker, 2008 ▶); cell refinement: *SAINT* (Bruker, 2008 ▶); data reduction: *SAINT*; program(s) used to solve structure: *SHELXS97* (Sheldrick, 2008 ▶); program(s) used to refine structure: *SHELXL97* (Sheldrick, 2008 ▶); molecular graphics: *X-SEED* (Barbour, 2001 ▶); software used to prepare material for publication: *publCIF* (Westrip, 2010 ▶).

## Supplementary Material

Crystal structure: contains datablocks I, global. DOI: 10.1107/S1600536810001145/hg2600sup1.cif

Structure factors: contains datablocks I. DOI: 10.1107/S1600536810001145/hg2600Isup2.hkl

Additional supplementary materials:  crystallographic information; 3D view; checkCIF report

## Figures and Tables

**Table 1:** Hydrogen-bond geometry (Å, °)

*D*—H⋯*A*	*D*—H	H⋯*A*	*D*⋯*A*	*D*—H⋯*A*
O3—H3⋯N2	0.84	1.86	2.600 (4)	146

**Fig. 1.**
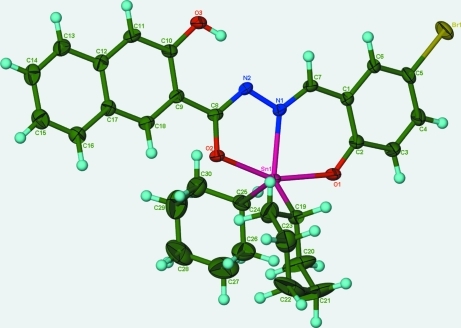
The molecular structure of [N'-(5-bromo-2-oxidobenzylidene-κO)-3-hydroxy-2- naphthohydrazidato-κ2N',O]dicyclohexyltin(IV) showing 50% probability displacement ellipsoids and the atom numbering. Hydrogen atoms are drawn as spheres of arbitrary radius.

## supplementary crystallographic information

### Experimental

The Schiff base ligand was prepared by the condensation reaction of 3-hydroxy-2-naphthoyl hydrazide with 5-bromosalicylaldehyde. The title compound was prepared from the equimolar reaction of the prepared Schiff base (0.74 g, 2.0 mmol) and dicyclohexyltin oxide (0.60 g, 2.0 mmol) in toluene. The solution was left for recrystallization for a week during which yellow crystals were obtained.

### Refinement

Hydrogen atoms were placed at calculated positions (C–H 0.95 to 0.98 Å) and were treated as riding on their parent carbon atoms, with *U*(H) set to 1.2–1.5 times *U*_eq_(C). The hydroxy-H was refined with a restraint of 0.84 ± 0.01 Å. There is a high peak near the tin atom.

### Crystal data

**Table:**

[Sn(C_6_H_11_)_2_(C_18_H_11_BrN_2_O_3_)]	*F*(000) = 1344
*M**_r_* = 668.18	*D*_x_ = 1.626 Mg m^−^^3^
Monoclinic, *P*2_1_/*c*	Mo *K*α radiation, λ = 0.71073 Å
Hall symbol: -P 2ybc	Cell parameters from 9975 reflections
*a* = 9.2442 (2) Å	θ = 2.2–30.4°
*b* = 9.9949 (2) Å	µ = 2.43 mm^−^^1^
*c* = 29.6493 (5) Å	*T* = 140 K
β = 94.874 (1)°	Block, yellow
*V* = 2729.54 (9) Å^3^	0.33 × 0.30 × 0.14 mm
*Z* = 4	

### Data collection

**Table:**

Bruker APEXII CCD area-detector diffractometer	4787 independent reflections
Radiation source: fine-focus sealed tube	4103 reflections with *I* > 2σ(*I*)
graphite	*R*_int_ = 0.027
ω scans	θ_max_ = 25.0°, θ_min_ = 1.4°
Absorption correction: multi-scan (*SADABS*; Sheldrick, 1996)	*h* = −10→10
*T*_min_ = 0.501, *T*_max_ = 0.727	*k* = −11→11
20482 measured reflections	*l* = −35→35

### Refinement

**Table:**

Refinement on *F*^2^	Primary atom site location: structure-invariant direct methods
Least-squares matrix: full	Secondary atom site location: difference Fourier map
*R*[*F*^2^ > 2σ(*F*^2^)] = 0.035	Hydrogen site location: inferred from neighbouring sites
*wR*(*F*^2^) = 0.079	H-atom parameters constrained
*S* = 1.09	*w* = 1/[σ^2^(*F*_o_^2^) + (0.030*P*)^2^ + 5.7701*P*] where *P* = (*F*_o_^2^ + 2*F*_c_^2^)/3
4787 reflections	(Δ/σ)_max_ = 0.001
335 parameters	Δρ_max_ = 1.60 e Å^−^^3^
1 restraint	Δρ_min_ = −0.49 e Å^−^^3^

### Special details

**Table:**

Geometry. All e.s.d.'s (except the e.s.d. in the dihedral angle between two l.s. planes) are estimated using the full covariance matrix. The cell e.s.d.'s are taken into account individually in the estimation of e.s.d.'s in distances, angles and torsion angles; correlations between e.s.d.'s in cell parameters are only used when they are defined by crystal symmetry. An approximate (isotropic) treatment of cell e.s.d.'s is used for estimating e.s.d.'s involving l.s. planes.
Refinement. Refinement of *F*^2^ against ALL reflections. The weighted *R*-factor *wR* and goodness of fit *S* are based on *F*^2^, conventional *R*-factors *R* are based on *F*, with *F* set to zero for negative *F*^2^. The threshold expression of *F*^2^ > σ(*F*^2^) is used only for calculating *R*-factors(gt) *etc*. and is not relevant to the choice of reflections for refinement. *R*-factors based on *F*^2^ are statistically about twice as large as those based on *F*, and *R*- factors based on ALL data will be even larger.

### Fractional atomic coordinates and isotropic or equivalent isotropic displacement parameters (Å^2^)

**Table:**

	*x*	*y*	*z*	*U*_iso_*/*U*_eq_	
Sn1	0.59441 (3)	0.67684 (3)	0.089632 (8)	0.02876 (9)	
Br1	1.11466 (6)	0.10654 (5)	0.162837 (16)	0.04959 (14)	
N1	0.7471 (3)	0.6588 (3)	0.14945 (9)	0.0258 (7)	
N2	0.7616 (3)	0.7713 (3)	0.17740 (10)	0.0285 (7)	
O1	0.6597 (3)	0.4803 (3)	0.07780 (9)	0.0414 (7)	
O2	0.5924 (3)	0.8642 (3)	0.12543 (8)	0.0324 (6)	
O3	0.8900 (3)	0.9235 (3)	0.23968 (9)	0.0402 (7)	
H3	0.8703	0.8519	0.2256	0.060*	
C1	0.8416 (4)	0.4343 (4)	0.13884 (12)	0.0268 (8)	
C2	0.7606 (4)	0.4016 (4)	0.09761 (12)	0.0318 (9)	
C3	0.7907 (5)	0.2795 (4)	0.07729 (14)	0.0426 (11)	
H3A	0.7378	0.2555	0.0496	0.051*	
C4	0.8943 (5)	0.1934 (4)	0.09617 (14)	0.0414 (10)	
H4	0.9133	0.1114	0.0816	0.050*	
C5	0.9709 (5)	0.2270 (4)	0.13655 (13)	0.0345 (9)	
C6	0.9463 (4)	0.3444 (4)	0.15792 (12)	0.0291 (9)	
H6	1.0001	0.3655	0.1858	0.035*	
C7	0.8270 (4)	0.5570 (4)	0.16245 (12)	0.0267 (8)	
H7	0.8819	0.5652	0.1909	0.032*	
C9	0.6882 (4)	0.9979 (4)	0.18743 (11)	0.0252 (8)	
C10	0.7946 (4)	1.0198 (4)	0.22456 (11)	0.0286 (8)	
C11	0.8030 (4)	1.1420 (4)	0.24509 (13)	0.0343 (9)	
H11	0.8773	1.1573	0.2687	0.041*	
C12	0.7055 (4)	1.2460 (4)	0.23257 (12)	0.0301 (9)	
C13	0.7133 (5)	1.3727 (4)	0.25347 (13)	0.0382 (10)	
H13	0.7882	1.3899	0.2767	0.046*	
C14	0.6160 (5)	1.4713 (4)	0.24113 (14)	0.0405 (10)	
H14	0.6235	1.5561	0.2556	0.049*	
C15	0.5039 (5)	1.4469 (4)	0.20675 (14)	0.0415 (10)	
H15	0.4342	1.5146	0.1988	0.050*	
C16	0.4952 (5)	1.3271 (4)	0.18494 (14)	0.0374 (9)	
H16	0.4203	1.3128	0.1614	0.045*	
C17	0.5959 (4)	1.2233 (4)	0.19668 (12)	0.0280 (8)	
C18	0.5929 (4)	1.0984 (4)	0.17489 (12)	0.0283 (8)	
H18	0.5216	1.0832	0.1504	0.034*	
C8	0.6791 (4)	0.8710 (4)	0.16197 (11)	0.0251 (8)	
C19	0.3711 (4)	0.6283 (4)	0.09615 (13)	0.0349 (9)	
H19	0.3689	0.5390	0.1113	0.042*	
C20	0.2916 (6)	0.6136 (7)	0.04918 (17)	0.0764 (19)	
H20A	0.3390	0.5432	0.0321	0.092*	
H20B	0.2973	0.6988	0.0324	0.092*	
C21	0.1328 (6)	0.5768 (9)	0.0527 (2)	0.103 (3)	
H21A	0.0816	0.5738	0.0220	0.124*	
H21B	0.1270	0.4868	0.0663	0.124*	
C22	0.0608 (6)	0.6752 (7)	0.0806 (2)	0.085 (2)	
H22A	−0.0408	0.6468	0.0832	0.102*	
H22B	0.0585	0.7633	0.0653	0.102*	
C23	0.1366 (5)	0.6897 (6)	0.12744 (19)	0.0605 (14)	
H23A	0.1297	0.6046	0.1442	0.073*	
H23B	0.0881	0.7603	0.1441	0.073*	
C24	0.2960 (5)	0.7263 (5)	0.12488 (17)	0.0487 (12)	
H24A	0.3027	0.8172	0.1119	0.058*	
H24B	0.3458	0.7274	0.1558	0.058*	
C25	0.6808 (5)	0.7552 (4)	0.03016 (13)	0.0371 (10)	
H25	0.7620	0.6946	0.0232	0.044*	
C26	0.5679 (6)	0.7490 (6)	−0.01051 (16)	0.0626 (14)	
H26A	0.4844	0.8069	−0.0049	0.075*	
H26B	0.5320	0.6561	−0.0146	0.075*	
C27	0.6336 (8)	0.7955 (7)	−0.05379 (17)	0.084 (2)	
H27A	0.7107	0.7323	−0.0611	0.101*	
H27B	0.5574	0.7952	−0.0794	0.101*	
C28	0.6953 (7)	0.9312 (7)	−0.0481 (2)	0.082 (2)	
H28A	0.6161	0.9958	−0.0442	0.098*	
H28B	0.7417	0.9564	−0.0758	0.098*	
C29	0.8062 (7)	0.9396 (6)	−0.0077 (2)	0.0762 (18)	
H29A	0.8397	1.0333	−0.0038	0.091*	
H29B	0.8913	0.8838	−0.0133	0.091*	
C30	0.7439 (6)	0.8921 (5)	0.03616 (16)	0.0553 (13)	
H30A	0.8219	0.8913	0.0612	0.066*	
H30B	0.6678	0.9552	0.0443	0.066*	

### Atomic displacement parameters (Å^2^)

**Table:**

	*U*^11^	*U*^22^	*U*^33^	*U*^12^	*U*^13^	*U*^23^
Sn1	0.03298 (16)	0.02502 (15)	0.02577 (14)	−0.00002 (11)	−0.01213 (10)	−0.00182 (11)
Br1	0.0606 (3)	0.0376 (3)	0.0485 (3)	0.0177 (2)	−0.0080 (2)	0.0070 (2)
N1	0.0310 (17)	0.0238 (17)	0.0212 (15)	−0.0046 (13)	−0.0053 (13)	−0.0007 (13)
N2	0.0329 (18)	0.0245 (17)	0.0263 (16)	−0.0037 (14)	−0.0078 (14)	−0.0043 (13)
O1	0.0531 (19)	0.0277 (15)	0.0383 (15)	0.0068 (13)	−0.0254 (14)	−0.0072 (12)
O2	0.0363 (16)	0.0266 (14)	0.0315 (14)	0.0019 (11)	−0.0141 (12)	−0.0044 (11)
O3	0.0490 (18)	0.0305 (16)	0.0369 (16)	0.0056 (13)	−0.0208 (13)	−0.0051 (12)
C1	0.031 (2)	0.024 (2)	0.0247 (18)	−0.0032 (16)	−0.0020 (16)	0.0026 (15)
C2	0.039 (2)	0.027 (2)	0.027 (2)	−0.0021 (17)	−0.0060 (17)	0.0013 (16)
C3	0.062 (3)	0.029 (2)	0.033 (2)	0.002 (2)	−0.018 (2)	−0.0063 (18)
C4	0.061 (3)	0.024 (2)	0.037 (2)	0.006 (2)	−0.007 (2)	−0.0030 (18)
C5	0.045 (2)	0.026 (2)	0.031 (2)	0.0057 (18)	−0.0014 (18)	0.0083 (17)
C6	0.036 (2)	0.023 (2)	0.0270 (19)	−0.0029 (16)	−0.0042 (16)	0.0045 (15)
C7	0.027 (2)	0.029 (2)	0.0230 (18)	−0.0057 (16)	−0.0052 (15)	0.0025 (15)
C9	0.026 (2)	0.029 (2)	0.0208 (17)	−0.0051 (16)	0.0018 (14)	0.0009 (15)
C10	0.033 (2)	0.031 (2)	0.0209 (18)	0.0002 (17)	−0.0036 (15)	−0.0003 (16)
C11	0.037 (2)	0.037 (2)	0.028 (2)	−0.0048 (18)	−0.0068 (17)	−0.0050 (17)
C12	0.036 (2)	0.030 (2)	0.0255 (19)	−0.0042 (17)	0.0067 (16)	−0.0026 (16)
C13	0.050 (3)	0.036 (2)	0.027 (2)	−0.005 (2)	0.0008 (19)	−0.0052 (18)
C14	0.058 (3)	0.031 (2)	0.035 (2)	0.001 (2)	0.014 (2)	−0.0080 (18)
C15	0.047 (3)	0.037 (2)	0.041 (2)	0.010 (2)	0.009 (2)	−0.002 (2)
C16	0.036 (2)	0.036 (2)	0.039 (2)	0.0025 (19)	0.0006 (18)	0.0014 (19)
C17	0.030 (2)	0.029 (2)	0.0263 (19)	−0.0028 (16)	0.0071 (16)	0.0012 (16)
C18	0.027 (2)	0.032 (2)	0.0254 (19)	−0.0038 (16)	−0.0007 (15)	−0.0006 (16)
C8	0.0224 (19)	0.032 (2)	0.0208 (18)	−0.0048 (16)	−0.0011 (15)	0.0014 (15)
C19	0.034 (2)	0.033 (2)	0.036 (2)	−0.0056 (18)	−0.0085 (18)	0.0028 (18)
C20	0.048 (3)	0.138 (6)	0.041 (3)	−0.035 (3)	−0.008 (2)	−0.012 (3)
C21	0.054 (4)	0.203 (9)	0.050 (3)	−0.063 (5)	−0.011 (3)	−0.016 (4)
C22	0.032 (3)	0.128 (6)	0.092 (5)	−0.011 (3)	−0.012 (3)	0.070 (4)
C23	0.044 (3)	0.056 (3)	0.082 (4)	−0.002 (2)	0.004 (3)	0.006 (3)
C24	0.041 (3)	0.041 (3)	0.064 (3)	−0.007 (2)	0.005 (2)	−0.002 (2)
C25	0.043 (3)	0.039 (2)	0.028 (2)	0.005 (2)	−0.0009 (18)	0.0019 (18)
C26	0.072 (4)	0.072 (4)	0.042 (3)	−0.009 (3)	−0.010 (3)	0.004 (3)
C27	0.100 (5)	0.117 (6)	0.033 (3)	−0.009 (4)	−0.002 (3)	0.006 (3)
C28	0.083 (4)	0.095 (5)	0.069 (4)	0.006 (4)	0.010 (3)	0.042 (4)
C29	0.095 (5)	0.061 (4)	0.076 (4)	−0.017 (3)	0.026 (4)	0.003 (3)
C30	0.066 (3)	0.050 (3)	0.050 (3)	−0.010 (3)	0.002 (2)	−0.003 (2)

### Geometric parameters (Å, °)

**Table:**

Sn1—O1	2.094 (3)	C16—C17	1.418 (6)
Sn1—C25	2.145 (4)	C16—H16	0.9500
Sn1—C19	2.145 (4)	C17—C18	1.405 (5)
Sn1—O2	2.153 (3)	C18—H18	0.9500
Sn1—N1	2.178 (3)	C19—C24	1.506 (6)
Br1—C5	1.910 (4)	C19—C20	1.525 (6)
N1—C7	1.296 (5)	C19—H19	1.0000
N1—N2	1.397 (4)	C20—C21	1.525 (7)
N2—C8	1.313 (5)	C20—H20A	0.9900
O1—C2	1.319 (5)	C20—H20B	0.9900
O2—C8	1.294 (4)	C21—C22	1.480 (10)
O3—C10	1.356 (4)	C21—H21A	0.9900
O3—H3	0.8400	C21—H21B	0.9900
C1—C6	1.404 (5)	C22—C23	1.507 (8)
C1—C2	1.416 (5)	C22—H22A	0.9900
C1—C7	1.425 (5)	C22—H22B	0.9900
C2—C3	1.399 (6)	C23—C24	1.527 (6)
C3—C4	1.371 (6)	C23—H23A	0.9900
C3—H3A	0.9500	C23—H23B	0.9900
C4—C5	1.380 (6)	C24—H24A	0.9900
C4—H4	0.9500	C24—H24B	0.9900
C5—C6	1.362 (5)	C25—C30	1.492 (6)
C6—H6	0.9500	C25—C26	1.527 (6)
C7—H7	0.9500	C25—H25	1.0000
C9—C18	1.367 (5)	C26—C27	1.537 (7)
C9—C10	1.429 (5)	C26—H26A	0.9900
C9—C8	1.475 (5)	C26—H26B	0.9900
C10—C11	1.364 (5)	C27—C28	1.476 (9)
C11—C12	1.404 (6)	C27—H27A	0.9900
C11—H11	0.9500	C27—H27B	0.9900
C12—C13	1.409 (6)	C28—C29	1.513 (8)
C12—C17	1.424 (5)	C28—H28A	0.9900
C13—C14	1.363 (6)	C28—H28B	0.9900
C13—H13	0.9500	C29—C30	1.540 (7)
C14—C15	1.411 (6)	C29—H29A	0.9900
C14—H14	0.9500	C29—H29B	0.9900
C15—C16	1.360 (6)	C30—H30A	0.9900
C15—H15	0.9500	C30—H30B	0.9900
			
O1—Sn1—C25	94.14 (15)	C24—C19—Sn1	113.4 (3)
O1—Sn1—C19	95.51 (14)	C20—C19—Sn1	109.4 (3)
C25—Sn1—C19	125.81 (15)	C24—C19—H19	107.4
O1—Sn1—O2	155.97 (9)	C20—C19—H19	107.4
C25—Sn1—O2	96.14 (14)	Sn1—C19—H19	107.4
C19—Sn1—O2	95.96 (13)	C19—C20—C21	110.7 (4)
O1—Sn1—N1	83.25 (10)	C19—C20—H20A	109.5
C25—Sn1—N1	116.02 (14)	C21—C20—H20A	109.5
C19—Sn1—N1	118.03 (13)	C19—C20—H20B	109.5
O2—Sn1—N1	72.71 (10)	C21—C20—H20B	109.5
C7—N1—N2	115.6 (3)	H20A—C20—H20B	108.1
C7—N1—Sn1	128.4 (2)	C22—C21—C20	111.0 (6)
N2—N1—Sn1	116.1 (2)	C22—C21—H21A	109.4
C8—N2—N1	112.4 (3)	C20—C21—H21A	109.4
C2—O1—Sn1	133.4 (2)	C22—C21—H21B	109.4
C8—O2—Sn1	115.3 (2)	C20—C21—H21B	109.4
C10—O3—H3	109.5	H21A—C21—H21B	108.0
C6—C1—C2	119.7 (3)	C21—C22—C23	112.6 (5)
C6—C1—C7	116.4 (3)	C21—C22—H22A	109.1
C2—C1—C7	123.9 (3)	C23—C22—H22A	109.1
O1—C2—C3	119.1 (3)	C21—C22—H22B	109.1
O1—C2—C1	123.3 (3)	C23—C22—H22B	109.1
C3—C2—C1	117.6 (3)	H22A—C22—H22B	107.8
C4—C3—C2	121.9 (4)	C22—C23—C24	110.6 (4)
C4—C3—H3A	119.0	C22—C23—H23A	109.5
C2—C3—H3A	119.0	C24—C23—H23A	109.5
C3—C4—C5	119.4 (4)	C22—C23—H23B	109.5
C3—C4—H4	120.3	C24—C23—H23B	109.5
C5—C4—H4	120.3	H23A—C23—H23B	108.1
C6—C5—C4	121.3 (4)	C19—C24—C23	111.5 (4)
C6—C5—Br1	119.4 (3)	C19—C24—H24A	109.3
C4—C5—Br1	119.3 (3)	C23—C24—H24A	109.3
C5—C6—C1	120.0 (3)	C19—C24—H24B	109.3
C5—C6—H6	120.0	C23—C24—H24B	109.3
C1—C6—H6	120.0	H24A—C24—H24B	108.0
N1—C7—C1	127.2 (3)	C30—C25—C26	111.4 (4)
N1—C7—H7	116.4	C30—C25—Sn1	114.0 (3)
C1—C7—H7	116.4	C26—C25—Sn1	110.9 (3)
C18—C9—C10	118.8 (3)	C30—C25—H25	106.7
C18—C9—C8	119.1 (3)	C26—C25—H25	106.7
C10—C9—C8	122.1 (3)	Sn1—C25—H25	106.7
O3—C10—C11	118.5 (3)	C25—C26—C27	110.8 (4)
O3—C10—C9	122.1 (3)	C25—C26—H26A	109.5
C11—C10—C9	119.3 (3)	C27—C26—H26A	109.5
C10—C11—C12	122.3 (3)	C25—C26—H26B	109.5
C10—C11—H11	118.9	C27—C26—H26B	109.5
C12—C11—H11	118.9	H26A—C26—H26B	108.1
C11—C12—C13	122.8 (4)	C28—C27—C26	111.0 (5)
C11—C12—C17	118.6 (3)	C28—C27—H27A	109.4
C13—C12—C17	118.6 (4)	C26—C27—H27A	109.4
C14—C13—C12	121.6 (4)	C28—C27—H27B	109.4
C14—C13—H13	119.2	C26—C27—H27B	109.4
C12—C13—H13	119.2	H27A—C27—H27B	108.0
C13—C14—C15	119.8 (4)	C27—C28—C29	111.8 (5)
C13—C14—H14	120.1	C27—C28—H28A	109.3
C15—C14—H14	120.1	C29—C28—H28A	109.3
C16—C15—C14	120.4 (4)	C27—C28—H28B	109.3
C16—C15—H15	119.8	C29—C28—H28B	109.3
C14—C15—H15	119.8	H28A—C28—H28B	107.9
C15—C16—C17	121.1 (4)	C28—C29—C30	112.0 (5)
C15—C16—H16	119.5	C28—C29—H29A	109.2
C17—C16—H16	119.5	C30—C29—H29A	109.2
C18—C17—C16	123.4 (4)	C28—C29—H29B	109.2
C18—C17—C12	118.1 (3)	C30—C29—H29B	109.2
C16—C17—C12	118.5 (4)	H29A—C29—H29B	107.9
C9—C18—C17	122.8 (3)	C25—C30—C29	110.6 (4)
C9—C18—H18	118.6	C25—C30—H30A	109.5
C17—C18—H18	118.6	C29—C30—H30A	109.5
O2—C8—N2	123.5 (3)	C25—C30—H30B	109.5
O2—C8—C9	118.6 (3)	C29—C30—H30B	109.5
N2—C8—C9	117.9 (3)	H30A—C30—H30B	108.1
C24—C19—C20	111.7 (4)		
			
O1—Sn1—N1—C7	0.0 (3)	C15—C16—C17—C18	−179.2 (4)
C25—Sn1—N1—C7	−91.3 (3)	C15—C16—C17—C12	1.2 (6)
C19—Sn1—N1—C7	92.7 (3)	C11—C12—C17—C18	−1.4 (5)
O2—Sn1—N1—C7	−179.8 (3)	C13—C12—C17—C18	177.5 (4)
O1—Sn1—N1—N2	179.7 (3)	C11—C12—C17—C16	178.3 (4)
C25—Sn1—N1—N2	88.4 (3)	C13—C12—C17—C16	−2.9 (5)
C19—Sn1—N1—N2	−87.6 (3)	C10—C9—C18—C17	0.7 (6)
O2—Sn1—N1—N2	0.0 (2)	C8—C9—C18—C17	−178.6 (3)
C7—N1—N2—C8	179.2 (3)	C16—C17—C18—C9	−178.1 (4)
Sn1—N1—N2—C8	−0.6 (4)	C12—C17—C18—C9	1.5 (6)
C25—Sn1—O1—C2	109.0 (4)	Sn1—O2—C8—N2	−1.3 (5)
C19—Sn1—O1—C2	−124.4 (4)	Sn1—O2—C8—C9	177.7 (2)
O2—Sn1—O1—C2	−6.2 (6)	N1—N2—C8—O2	1.3 (5)
N1—Sn1—O1—C2	−6.8 (4)	N1—N2—C8—C9	−177.7 (3)
O1—Sn1—O2—C8	0.1 (4)	C18—C9—C8—O2	7.8 (5)
C25—Sn1—O2—C8	−114.7 (3)	C10—C9—C8—O2	−171.5 (3)
C19—Sn1—O2—C8	118.2 (3)	C18—C9—C8—N2	−173.2 (3)
N1—Sn1—O2—C8	0.7 (2)	C10—C9—C8—N2	7.5 (5)
Sn1—O1—C2—C3	−171.0 (3)	O1—Sn1—C19—C24	153.9 (3)
Sn1—O1—C2—C1	8.8 (6)	C25—Sn1—C19—C24	−106.9 (3)
C6—C1—C2—O1	179.4 (4)	O2—Sn1—C19—C24	−4.9 (3)
C7—C1—C2—O1	−2.4 (6)	N1—Sn1—C19—C24	68.6 (3)
C6—C1—C2—C3	−0.7 (6)	O1—Sn1—C19—C20	−80.7 (4)
C7—C1—C2—C3	177.5 (4)	C25—Sn1—C19—C20	18.4 (4)
O1—C2—C3—C4	179.9 (4)	O2—Sn1—C19—C20	120.4 (4)
C1—C2—C3—C4	0.1 (7)	N1—Sn1—C19—C20	−166.0 (4)
C2—C3—C4—C5	0.5 (7)	C24—C19—C20—C21	−54.5 (7)
C3—C4—C5—C6	−0.4 (7)	Sn1—C19—C20—C21	179.1 (5)
C3—C4—C5—Br1	179.8 (4)	C19—C20—C21—C22	55.3 (8)
C4—C5—C6—C1	−0.2 (6)	C20—C21—C22—C23	−56.7 (7)
Br1—C5—C6—C1	179.6 (3)	C21—C22—C23—C24	55.8 (7)
C2—C1—C6—C5	0.8 (6)	C20—C19—C24—C23	54.3 (5)
C7—C1—C6—C5	−177.5 (4)	Sn1—C19—C24—C23	178.4 (3)
N2—N1—C7—C1	−175.0 (3)	C22—C23—C24—C19	−54.0 (6)
Sn1—N1—C7—C1	4.7 (6)	O1—Sn1—C25—C30	−143.8 (3)
C6—C1—C7—N1	173.8 (4)	C19—Sn1—C25—C30	116.3 (3)
C2—C1—C7—N1	−4.4 (6)	O2—Sn1—C25—C30	14.4 (3)
C18—C9—C10—O3	178.2 (4)	N1—Sn1—C25—C30	−59.3 (4)
C8—C9—C10—O3	−2.5 (6)	O1—Sn1—C25—C26	89.5 (3)
C18—C9—C10—C11	−3.1 (6)	C19—Sn1—C25—C26	−10.4 (4)
C8—C9—C10—C11	176.2 (4)	O2—Sn1—C25—C26	−112.3 (3)
O3—C10—C11—C12	−177.9 (4)	N1—Sn1—C25—C26	174.0 (3)
C9—C10—C11—C12	3.4 (6)	C30—C25—C26—C27	56.1 (6)
C10—C11—C12—C13	−179.9 (4)	Sn1—C25—C26—C27	−175.8 (4)
C10—C11—C12—C17	−1.1 (6)	C25—C26—C27—C28	−56.2 (7)
C11—C12—C13—C14	−179.0 (4)	C26—C27—C28—C29	55.7 (8)
C17—C12—C13—C14	2.2 (6)	C27—C28—C29—C30	−54.9 (8)
C12—C13—C14—C15	0.2 (6)	C26—C25—C30—C29	−54.8 (6)
C13—C14—C15—C16	−2.0 (6)	Sn1—C25—C30—C29	178.8 (4)
C14—C15—C16—C17	1.3 (6)	C28—C29—C30—C25	54.1 (7)

### Hydrogen-bond geometry (Å, °)

**Table:**

*D*—H···*A*	*D*—H	H···*A*	*D*···*A*	*D*—H···*A*
O3—H3···N2	0.84	1.86	2.600 (4)	146
